# Spectral Imaging at the Microscale and Beyond

**DOI:** 10.3390/s140508162

**Published:** 2014-05-06

**Authors:** François Paquet-Mercier, Jesse Greener

**Affiliations:** Département de Chimie, Université Laval, 1045 Avenue de la Médecine, Québec, QC G1V 0A6, Canada; E-Mail: francois.paquet-mercier.1@ulaval.ca

**Keywords:** spectral imaging, multispectral imaging, chemical imaging, imaging spectroscopy, multi-band imaging, hyperspectral imaging, spectroscopy, microscopy, mapping

## Abstract

Here we give context to the special issue “Spectral Imaging at the Microscale and Beyond” in *Sensors*. We start with an introduction and motivation for the need for spectral imaging and then present important definitions and background concepts. Following this, we review new developments and applications in environmental monitoring, biomaterials, microfluidics, nanomaterials, healthcare, agriculture and food science, with a special focus on the articles published in the special issue. Some concluding remarks put the presented developments in context vis-à-vis the future of spectral imaging.

## 1. Introduction

Never before has the demand for information intensive characterization with high spatial resolution been so high. Traditionally, small-scale imaging was conducted by optical or electron microscopes, or by scanning probe techniques, such as atomic force microscopy. However, sustained advances in fields such as microfluidics, materials sciences, microbiology and food sciences require more information per pixel than traditional imaging techniques. Spectroscopy is a general concept, which involves analyzing broad-band electromagnetic fields (which are either radiating photons or near-field electromagnetic fields) after their interaction with matter. Due to the many mechanisms of interaction, spectroscopy can provide profound insights into analyte properties, including molecular vibrations, electronic transitions and nuclear precession rates about an external magnetic field. All of this information is available without the need for specific sample preparation or the addition of probe materials. Moreover, spectroscopy is generally passive, enabling consecutive, real-time measurements of analytes as they evolve in time. However, typically, spectroscopy does not yield spatially resolved measurements. Therefore, spectral imaging techniques have been developed to enable spatially-resolved spectroscopic characterization. We note that spectral imaging is known in different disciplines by various terms, such as: Multispectral imaging, chemical imaging, imaging spectroscopy, multi-band imaging or hyperspectral imaging.

The special issue of *Sensors* “Spectral Imaging at the Microscale and Beyond” was launched in response to the rapid pace of new technical developments and applications in spectral imaging. This short editorial outlines some basic concepts as a compliment to the special issue and then highlights the published work therein. We conclude the article with some summary comments and suggestions for future work.

## 2. Definitions

True spectral imaging should include techniques that quantitatively analyze intensity and wavelength of the electromagnetic spectrum. Therefore, we exclude simple colour photography because, while colour images appear to capture all visible wavelengths, in fact these colours are usually generated by a mixture of only three colour bands (red, green, blue). Similarly, thermal imaging is not a considered a spectroscopic technique because the light intensity is averaged over a wide-spectral window, which discards resolution in the spectral domain. Contributors to this edition collected spatially-resolved spectroscopic information from UV-visible and near-infrared (NIR) in both transmission [1] and surface reflectance configurations [2,3], fluorescence [3,4] and inelastic scattering (Raman spectroscopy) [5,6]. Other spectral modes that are sometimes used for imaging include infrared and magnetic resonance. Secondly, we specify the imaging component of the technique as being zero-dimensional (point imaging), one-dimensional (linear imaging), two-dimensional (area imaging) or three-dimensional (volume imaging). Higher dimensionality spectral maps are typically constructed from lower-order image data. For example, two-dimensional Raman spectral maps can be built by generating zero-dimensional spectra, point-by-point, *via* sequential acquisition of spectra originating from a laser excitation source as it scans over the sample. Spectral imaging, is therefore, an information intensive approach that generates maps with each pixel reporting on a broad range of analyte properties, depending on the spectroscopic mode. Technical developments are continuously being made to address current limitations. For example, contributors in this special edition developed a method for the reconstruction of multispectral image from RGB image files of reflected light, thereby opening the way for utilization of regular colour cameras for spectral imaging [7]. Other technical developments in this special issue include algorithms for multivariate data analysis [8] and data preprocessing to minimize the difficulty of management and processing of large data files [9].

## 3. Applications

Development and utilization of spectral imaging requires a multi-disciplinary approach, bringing together physics, chemistry, instrument development and concepts in applied mathematics related to data analysis and data management. The applications are even more varied. The contributions to this special issue covered a range of applications, including environmental monitoring [1,9], biological materials [2–5], microfluidics [5], nanomaterials [6], healthcare [7] and agriculture and food science [8,10]. For example, in the paper of Mehrubeoglu *et al.*, a method based on spectral imaging in the visible and near-infrared spectrum was presented for the analysis of the types and abundances of different algae that are implicated in natural algal blooms [1]. Bertani *et al.*, presented a confocal microscope for visible and near-infrared spectroscopic measurements in reflectance mode using supercontinuum laser source with sub-micrometer spatial resolution [2]. This system allowed the acquisition of three-dimensional spectral images of human melanoma cells. The spectra reported on scattering and light absorption related to electronic and vibrational transitions, which were used in the recognition of cell organelles. Hirvonen *et al.*, also presented a setup for spectral imaging of visible and near-infrared in reflectance and luminescence modes as a practical alternative for fast imaging over a broad spectral range [3]. The spatial resolution of 250 μm for this system has many potential applications for large-area samples. Moreover, it was widely customizable, leading to possibilities for enhanced spectral resolution and operation in transmission mode. Annamdevula *et al.*, developed a method to compare the performance of wide-field fluorescence and confocal fluorescence microscopes in terms of their acquisition parameters (light intensity, optics and detector properties) and their spectral images produced. The results were compared in a photo bleaching study of of green fluorescent proteins (GFP) expressed by pulmonary microvascular endothelial cells from rats [4]. The approach should be useful for the optimization of fluorescence spectral imaging systems for different applications. Huang *et al.* reviewed recent advances in visible and near-infrared spectroscopic imaging in transmission, reflectance and fluorescence modes for the assessment of food quality and safety [10]. In their review, the authors made a comprehensive summary of the different types of food products that have recently been studied by spectral imaging, the different image processing methods implemented and the models used to interpret the data.

In addition to visible, near-infrared and fluorescence spectroscopic modes discussed above, Raman spectral imaging is an excellent tool, in part due to the highly focused excitation laser, which allows spatial resolution to approach the diffraction limit. In addition, its measurements of vibrational bands result in the generation of rich chemical information. However, the drawback is its low signal intensity, which leads to long data acquisition times, particularly for spectral maps which are comprized of many point measurements. In their paper, Paquet-Mercier *et al.* used surface enhanced Raman spectroscopy (SERS) at nanostructured metallic surfaces to get strong signal intensities within microfluidic channels [5]. Microfluidic flows were used to attain precise control over the chemical and physical environment of biofilm growth cells. In this paper, the authors showed the possibility to make *in situ* measurements of biofilm precursor materials in relevant concentrations (low millimolar range). This allowed the generation of one-dimensional spectral images of flow cross-sections containing a biofilm precursor material in flow-patterning conditions. The SERS enhancement was also used by Kazemi-Zanjani *et al.* However, they measured the signal very close to the SERS surface (in the near-field) in order to overcome diffraction limitations [6]. By using a metallized atomic force microscopy (AFM) tip as their SERS surface, they were able to specify the measurement location along the length of a single silicon nanowire to obtain Raman spectral images with spatial resolution on the order of nanometers. The authors demonstrated the ability to measure differences in straight and bent parts of the silicon nanowire *via* frequency shifts and changes to peak widths in the Raman bands.

Fast, accurate and cost-effective methods for spectral imaging are of great importance. Therefore, this special issue of Sensors also contains contributions that demonstrate new methods for data analysis to obtain more precise and meaningful information from spectral images. For example, in the paper presented by Kong *et al.*, an algorithm for fast multivariate data analysis from near-infrared two-dimensional spectral images of rice seeds was developed for the identification of their cultivars [8]. A paper by Nishidate *et al.*, demonstrated a method based on the Wiener estimation for the reconstruction of visible wavelength spectral information from RGB data files that were generated from a regular colour camera [7]. This development opens the way for the use of low-cost detection options while maintaining good accuracy. With this system, the authors demonstrated the ability to make measurements of melanin and hemoglobin content from of skin and tissue-like agar gel samples. Spectral imaging is often used for the detection and characterization of land and ocean surfaces using data collected from airplanes and satellites. In this issue, Gao and Liu demonstrated a fast smoothing algorithm to remove the atmospheric gases and aerosols from these 2D images in order to retrieve quantitative information [9].

## 4. Conclusions

Spectral imaging is becoming more widely used than ever before, with current developments relying on a strong commitment to multidisciplinary. Spectral imaging is, therefore, highly customizable and versatile, leading to many unprecedented and creative applications. At the same time, the necessary complexity inherent in its development and utilization also presents a roadblock to wider utilization. In this respect, this special edition provides examples of applications that are both cutting-edge and highly practical. Moving forward, we believe that the community of users and developers should complement their new work with systematic reviews outlining application of spectral imaging in different areas. This will allow the wider scientific community to appreciate the vast potential of spectral imaging to enable new discoveries at the microscale and beyond.
